# Bronchoscopist's perception of the quality of the single-use bronchoscope (Ambu aScope4™) in selected bronchoscopies: a multicenter study in 21 Spanish pulmonology services

**DOI:** 10.1186/s12931-020-01576-w

**Published:** 2020-12-02

**Authors:** Javier Flandes, Luis Fernando Giraldo-Cadavid, Javier Alfayate, Iker Fernández-Navamuel, Carlos Agusti, Carmen M. Lucena, Antoni Rosell, Felipe Andreo, Carmen Centeno, Carmen Montero, Iria Vidal, Lucía García-Alfonso, Antonio Bango, Miguel Ariza, Rocío Gallego, Marta Orta, Salvador Bello, Elisa Mincholé, Alfons Torrego, Virginia Pajares, Héctor González, Aurelio Luis Wangüemert, Julio Pérez-Izquierdo, Carlos Disdier, Blanca de Vega Sanchez, Rosa Cordovilla, Juan Cascón, Antonio Cruz, J. Javier García-López, Luis Puente, Paola Benedetti, Cristina L. García-Gallo, Gema Díaz Nuevo, Silvia Aguado, Concepción Partida, Prudencio Díaz-Agero, Estefanía Luque Crespo, María Pavón, Francisco Páez, Enrique Cases, Raquel Martínez, Andrés Briones, Cleofe Fernández, Concepción Martín Serrano, Ana Maria Uribe-Hernández, Jose Robles

**Affiliations:** 1 Pneumology Service, Bronchoscopy and Interventional Pulmonology Unit, University Hospital “Fundación Jiménez Díaz”, ISS-FJD CIBERES, Madrid, Spain; 2 Interventional Pulmonology, Fundación Neumológica Colombiana, Profesor Titular Universidad de La Sabana, Bogotá, DC Colombia; 3 Pneumology Service, Hospital Clínic Universitari, Barcelona, Spain; 4 Pneumology Service, Hospital Universitari de Bellvitge, Barcelona, Spain; 5 Pneumology Service, Hospital Universitari Germans Trias i Pujol, Badalona, Barcelona, Spain; 6 Pulmonology Service, Complexo Hospitalario Universitario, A Coruña, Spain; 7 Pneumology Service, Central University Hospital of Asturias, Oviedo Asturias, Spain; 8 Pneumology Service, Hospital San Pedro de Alcántara, Cáceres, Spain; 9 Pneumology Service, Miguel Servet University Hospital, Zaragoza, Spain; 10 Pneumology Service, Hospital de la Santa Creu i Sant Pau, Barcelona, Spain; 11 Pneumology Service, University Hospital of the Canary Islands, Santa Cruz de Tenerife, Spain; 12 Pneumology Service, Hospital San Juan de Dios, Santa Cruz de Tenerife, Spain; 13 Pneumology Service, Galdakao University Hospital, Bilbao Vizcaya, Spain; 14 Pneumology Service, Hospital Clínico Universitario, Valladolid, Spain; 15 Pneumology Service, University Assistance Complex, Salamanca, Spain; 16 Pneumology Service, Hospital Universitario Gregorio Marañón, Madrid, Spain; 17 Pneumology Service, Puerta de Hierro University Hospital, Madrid, Spain; 18 Thoracic Surgery Service, Hospital Universitario La Paz, Madrid, Spain; 19 Pneumology Service, Virgen de Macarena University Hospital, Seville, Spain; 20 Pneumology Service, Carlos Hay Regional University Hospital, Malaga, Spain; 21 Respiratory Endoscopy Unit, Hospital Universitari i Politècnic La Fe, Valencia, Spain; 22 Pneumology Service, General University Hospital, Alicante, Spain; 23 Fundación Neumológica Colombiana, Bogotá, DC Colombia; 24 Industrial Electronic Engineering, GHS SL, Madrid, Spain; 25 Department of Epidemiology and Biostatistics, School of Medicine, Universidad de La Sabana, Autonorte de Bogota Km 7, La Caro, Chía, Colombia

**Keywords:** Bronchoscopy, Quality, CUSUM analysis, aScope4™, Bronchoalveolar lavage

## Abstract

**Background:**

The disposable bronchoscope is an excellent alternative to face the problem of SARS-CoV-2 and other cross infections, but the bronchoscopist's perception of its quality has not been evaluated.

**Methods:**

To evaluate the quality of the Ambu-aScope4 disposable bronchoscope, we carried out a cross-sectional study in 21 Spanish pulmonology services. We use a standardized questionnaire completed by the bronchoscopists at the end of each bronchoscopy. The variables were described with absolute and relative frequencies, measures of central tendency and dispersion depending on their nature. The existence of learning curves was evaluated by CUSUM analysis.

**Results:**

The most frequent indications in 300 included bronchoscopies was bronchial aspiration in 69.3% and the median duration of these was 9.1 min. The route of entry was nasal in 47.2% and oral in 34.1%. The average score for ease of use, image, and aspiration quality was 80/100. All the planned techniques were performed in 94.9% and the bronchoscopist was satisfied in 96.6% of the bronchoscopies. They highlighted the portability and immediacy of the aScope4TM to start the procedure in 99.3%, the possibility of taking and storing images in 99.3%. The CUSUM analysis showed average scores > 70/100 from the first procedure and from the 9th procedure more than 80% of the scores exceeded the 80/100 score.

**Conclusions:**

The aScope4™ scored well for ease of use, imaging, and aspiration. We found a learning curve with excellent scores from the 9th procedure. Bronchoscopists highlighted its portability, immediacy of use and the possibility of taking and storing images.

## Background

Fiberoptic bronchoscopy is a widely used procedure in most hospitals around the world, especially for the diagnosis of infectious, inflammatory and tumor lung diseases. It is estimated that 92,000 bronchoscopies are performed annually in Spain, with a tendency to growth. Unfortunately, bronchoscopy can spread infections by spreading an infection in the same patient, by cross-infection between patients, or by infecting the personnel involved in the procedure [1].

The effects of cross infection can be severe for the patient and the health system and include complications of the infection and its consequences in terms of laboratory tests, medications, hospital stay, disability, and direct and indirect costs. For this reason, it is necessary to take extreme care and precautions in the decontamination and cleaning of equipment [2]. Despite complex and advanced endoscope cleaning and disinfection systems, disinfection is often inadequate [3–5], with the consequent risk of cross infection [5–7].

A disposable bronchoscope could decrease the risk of cross infection and increase accessibility to bronchoscopy in centers of less complexity or with limited resources and has been recommended by most respiratory societies for bronchoscopies during the SARS-COv-2 pandemic [8–11]. Patients on mechanical ventilation [12], immunosuppressed or with infectious contagious diseases are those with the highest risk for cross infections and where these devices may have a more relevant role. These bronchoscopes are also desirable for procedures with a high risk of damage the bronchoscope (e.g. bronchoscopy by orotracheal tube), to reduce repair costs [13].

The utility of single-use bronchoscopes has been extensively studied in anesthesiology, where they have been compared with reusable bronchoscopes in terms of ease of use [14]. In the field of pulmonology, there are not publications listing the perception of pulmonologists when using these bronchoscopes for conventional diagnostic and therapeutic techniques.

This study seeks to evaluate the perception of the bronchoscopist about the quality of the Ambu^®^ aScope4™ bronchoscope and the existence of a learning curve during the performance of conventional bronchoscopies of low complexity in the usual practice in pulmonology services of university hospitals of third level of care.

## Methods

### Design

A prospective, observational, multicenter, cross-sectional study was conducted of an approved disposable bronchoscope (Ambu^®^ aScope4™) with European certification (CEA) and used according to the product data sheet.

The study was carried out between February and August 2018, in tertiary care university hospitals with experience in performing bronchoscopies. The study was approved by the ethics committees of each institution and all the participants signed the informed consent both for their participation in the study and for the bronchoscopy.

The inclusion criteria were patients over 18 years with indication for the performance of a diagnostic bronchoscopy and that the procedure be approved by signing the informed consent. Exclusion criteria were bronchoscopies that required flexible interventional bronchoscopies or in which highly complex equipment was to be used. 300 subjects were recruited prospectively and consecutively in 21 Spanish pulmonology services. Bronchoscopists had an experience of more than 500 bronchoscopies.

There is few published experience with this bronchoscope for complex techniques, thus cases with risk of bleeding were excluded from the study. The bronchoscopies were only diagnostic bronchoscopies with bronchial aspiration and bronchoalveolar lavage (BAL) and therapeutic bronchoscopies that involved the aspiration of secretions or hematic remains.

The procedures were performed following the same sedation and analgesia protocol as in bronchoscopies with the conventional videobronchoscope: local anesthesia on the airway with 1% lidocaine. On the other hand, conscious sedation with propofol, fentanyl or midazolam was used at the discretion of each research center who participates in the study. The bronchial aspirate (BAS) consisted of collecting the secretions found during the examination by means of simple aspiration or by using instillations of small volumes of physiological serum (5–10 cc) to favor the collection or try to carry away cellularity or microbiota. of the airway.

BAL was performed by interlocking in a segmental or subsegmental bronchus (depending on the bronchial anatomy), three 50-cc aliquots of 0.9% saline were instilled and successively aspirated and processed for study. It was assumed that the first aliquot would collect a sample from the bronchial area, while the last volume instilled would carry distal airway and alveolar contents.

The study aims to validate the bronchoscope by means of a personal subjective questionnaire carried out by an experienced bronchoscopist without considering quantifiable parameters such as the quality of the samples or the volume of the BAL recovered.

In the cases included in the study, the bronchoscopy was performed by two observers, the main operator who operated the bronchoscope and a collaborator who analyzed the exploration during its development. A video of each bronchoscopy was recorded and saved in the memory of the Ambu^®^ aView™ device for later visualizations. The videos were labeled according to the order number and date of the examination, without the patient data being included. All procedures were anonymous in storage and confidentiality and privacy regulations were respected.

A search of the literature was carried out on questionnaires that measured the quality of the fiberoptic bronchoscopes for the performance of diagnostic bronchoscopies and none was found, so a new questionnaire was constructed on the Bronchoscope Quality Questionnaire (BQQ) by means of an expert consensus to establish the relevant items and domains to evaluate in the quality of the bronchoscope. A pilot test was carried out to adjust the questions and the psychometric measures of the final instrument were evaluated.

The BQQ was assessed independently and masked by the two bronchoscopists after the bronchoscopy at two different times. Both questionnaires were archived and after a period of 1–15 days from the bronchoscopy, one of the two bronchoscopists viewed the video of the bronchoscopy, and completed the questionnaire again.

## Statistic analysis

The qualitative variables were described with absolute and relative frequencies, the quantitative ones by means and standard deviations, or medians and interquartile range (25th percentile to 75th percentile) depending on their distribution. The internal consistency of the questionnaire was measured with the Cronbach's alpha coefficient. To unify the item scores and to be able to compare them, the standardized scores were calculated using the following equation:

$$\frac{{\text{score obtained on the item}}}{{\text{maximum possible score on the item}}} \times 100.$$

In this way, the standardized score was left with a range from 0 (worst possible score) to 100 (best possible score).

To assess the bronchoscopies quality related to the number of procedures performed with the aScope4 and the existence of learning curves, we used the binary CUSUM analysis method. We describe the details of this method in the Additional file 1, which we summarize below. We consider an acceptable failure rate $({p}_{0})$ 10% (90% of the scores in the evaluated aspect ≥ 80/100) and an unacceptable failure rate of 20% $({p}_{1})$ (less than 80% of the scores in the evaluated aspect ≥ 80/100); we defined a type I error (probability of falsely qualifying the bronchoscope as inadequate, designated as α) of 0.1 and a type II error (probability of falsely qualifying the bronchoscope as excellent, designated as β) of 0.1 [15–17]. We plotted the CUSUM graph by plotting the index number of each case (bronchoscopy) on the x-axis versus the cumulative sum score after that case on the y-axis. Consecutive failures drive the CUSUM curve upward while consecutive successes drive the CUSUM curve downward.

The CUSUM chart includes horizontal lines called decision limits (h_1_ y h_0_), which are the limits of an acceptable or unacceptable error rate. When the CUSUM curve crosses a decision boundary from above, it is inferred that the failure rates were within the predetermined acceptable rate of 10% (excellent performance); when the CUSUM curve crosses a decision limit from below, it is inferred that failure rates have reached the unacceptable failure rate of 20% (inadequate performance); If the CUSUM curve is stable between two decision limits, stable performance is inferred within good levels. Therefore, good performance is assumed when the CUSUM curve slopes downward or remains stable, but when the curve slopes upward it indicates a lower than acceptable success rate. Decision limits (h1 and h0) were calculated based on the risk of type I errors (α) and II (β). In our case, as $\alpha =\beta$ = 0,1; ${p}_{0}$ = 10% and ${p}_{1}$ = 20%; therefore h_0_ = h_1_ = 2,71. For this reason, we mark the decision limits of our CUSUM charts as horizontal lines starting from the axis and at intervals of 2.71. Software used Microsoft Excel 2016 (Microsoft Corporation, Redmond, WA, USA) and STATA vs 14.0 (StataCorp, Texas, USA).

Assuming a confidence of 95%, a margin of error of 6% and an average proportion of 50%

for the qualitative variables (more demanding scenario in terms of sample size), a sample size of 267 bronchoscopies was deemed necessary for the study, to compensate possible losses it was decided to increase to 300 bronchoscopies.

## Results

A total of 300 bronchoscopies were performed, 15 procedures were made by each Spanish pulmonology services, 36 bronchoscopist participated with a median of 4 bronchoscopies (IQR 2.1—8.4)**.** The most frequent indications were BAS in 69.3% of all cases (208/300; 95% CI 63.9–74.3%) and BAL in 125 of all bronchoscopies (41.7%; 95% CI: 36.2–47.3%) (Table 1 and Additional file 1: Fig. S1). The nasal route of entry was used in 47.2% (141/300; 95% CI 41.6–52.8%) and the oral one in 34.1% of cases (95% CI 29.0–39.7%) (Table 1 and Additional file 1: Fig. S2). The duration of the bronchoscopy had a median of 9.1 min (IQR 6.0–13.0) (Table 1). The reliability of the questionnaire measured by Cronbach's alpha was 0.88.

**Table 1:** General characteristics

	N	%	95% CI	
Indication for bronchoscopy				
Bronchial lavage or bronchial aspirate	208	69.3	63.9%	74.3%
Bronchoalveolar lavage	125	41.7	36.2%	47.3%
Therapeutic aspiration of secretions	30	10.0	7.1%	13.9%
Bronchial biopsy	17	5.7	3.6%	8.9%
Route of entry for bronchoscopy				
Nasal	141	47.2	41.6%	52.8%
Oral	102	34.1	29.0%	39.7%
Orotracheal tube	27	9.0	6.3%	12.8%
Tracheostomy	26	8.7	5.1%	14.6%
VMNI mask	1	0.3	0.1%	1.9%
Other	2	0.7	0.2%	2.4%
Bronchoscopy duration				
Time in minutes, median (IQR)	9.1 (6.0–13.0)			

*95% CI* 95% confidence interval, *IQR* interquartile range

**Fig. 1:**
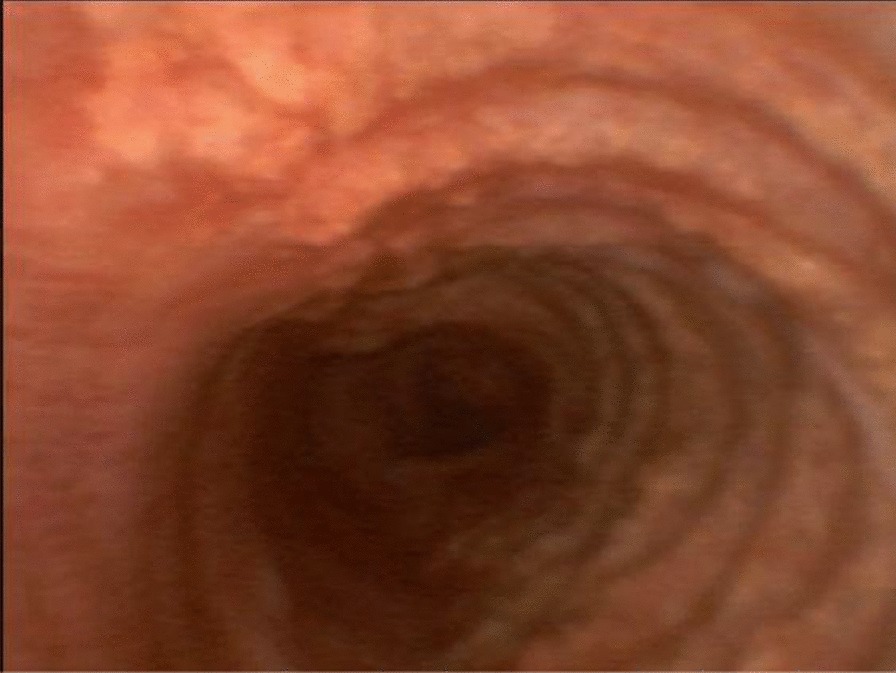
Image of the proximal third of the trachea obtained with aScope 4 of a patient showing an osteochondroplastic tracheobroncopathy

The average in user-friendliness, image and aspiration quality was 4 out of a maximum score of 5 (standardized score: 80/100; for a maximum score of 100 and a minimum of 0). The average standardized score for ease of use, image quality, and aspiration was 80/100 (Table 2 and Fig. 1). In 6% of the cases it was necessary to change the aScope 4, the most frequent reasons were limitation to reach the goals of the procedure and damage to the bronchoscope. 54.4% considered that the aScope had lower image quality than reusable video endoscopes. In more than 90% of the cases, all the pulmonary segments could be reached and all the planned techniques could be performed, for a general satisfaction with the device of 86.4% and a recommendation for its use in similar cases in 86.4% of the cases. times (Table 3).

**Table 2:** Quality of the aScope 4 bronchoscope

		Median	IQR^a^	Score standardized (%)^b^
Complexity to assemble the device	0: impossible → 5: extremely easy	4.0	4.0–5.0	80
Intubation facility	0: extremely difficult → 10: extremely easy	8.0	8.0–9.0	80
Ease of maneuvering in the tracheobronchial tree	0: extremely difficult → 10: extremely easy	8.0	7.0–9.0	80
Vasculature image quality	0: impossible to see vasculature → 5: maximum sharpness of vasculature	4.0	3.0–4.0	80
Mucous image quality	0: impossible to see mucosa folds → 5: maximum clarity of mucosa folds	4.0	3.0–4.0	80
Image quality of subsegmental bronchi from the segmental bronchus	0: impossible to see subsegmental bronchi → 5: maximum sharpness of subsegmental bronchi	4.0	3.0–4.0	80
Image quality for pathological mucosal alterations	0: impossible visualization of pathological lesions → 5: maximum sharpness of pathological lesions	4.0	3.0–4.0	80
Image quality in case of bleeding	0: complete image loss in bleed → 4: highest image quality in bleed	3.0	2.0–3.0	75
Global image quality	0: no image → 10: optimal image quality	8.0	7.0–8.0	80
Quality to suction secretions	0: impossible to suction secretions → 5: excellent ability to suction secretions	4.0	4.0–4.0	80
Quality to suction blood clots and debris	0: impossible to suction blood clots and debris → 5: excellent ability to suction clots and blood debris	4.0	4.0–4.0	80
Capacity to suction blood in active bleeding	0: impossible to suction blood in active bleeding → 5: excellent ability to suction blood in active bleeding	4.0	3.0–4.0	80
Global suction quality	0: zero suction capacity → 10: optimal suction capacity	8.0	8.0–9.0	80
Average score for ease of use, image quality and aspiration quality				80

*95% CI* 95% confidence interval

^a^IQR: interquartile range

^b^Standardized score: calculated by dividing the score obtained by the maximum possible score and multiplying by 100, the best possible standardized score is 100% and the worst is 0%

**Table 3:** Capacity of the aScope 4TM bronchoscope to perform the planned techniques

		n	%	95% CI
Loss of functionality or deterioration during the procedure	No	287	97.0	94.3–98.4%
	Yes	9	3.0	1.6–5.7%
Need to change bronchoscope during the procedure	It was not necessary to change	283	94.3	91.1–96.4%
	Changed due to rupture or damage to the bronchoscope	2	0.7	0.2–2.4%
	Changed for bad aspiration	1	0.3	0.1–1.9%
	Changed for limitation to reach procedure goals	3	1.0	0.3–2.9%
	It was changed for a bad image	1	0.3	0.1–1.9%
	It was changed for another reason	10	3.3	1.8–6.0%
Compared to other video-endoscopic equipment how did you find the Ambú	Much better quality	9	3.1	1.6–5.7%
	More quality	35	11.9	8.7–16.1%
	Equal quality	90	30.6	25.6–36.1%
	Less quality	152	51.7	46.0–57.4%
	Much less quality	8	2.7	1.4–5.3%
Were you able to reach all lung segments?	Yes	272	91.9	88.2–94.5%
	No	19	6.4	4.1–9.8%
	Does not apply	5	1.7	0.7–3.9%
Ability to perform all the techniques provided	Yes	280	94.9	91.8–96.9%
	No	12	4.1	2.3–7.0%
	Does not apply	3	1.0	0.3–2.9%
General satisfaction with the bronchoscope	Very satisfied	3. 4	11.6	8.4–15.7%
	Satisfied	102	34.7	29.5–40.3%
	Neutral	118	40.1	34.7–45.8%
	Somewhat unsatisfied	39	13.3	9.9–17.6%
	Dissatisfied	one	0.3	0.1–1.9%
I would recommend using this bronchoscope for similar procedures	I would recommend that it always be used	3. 4	11.6	8.4–15.7%
	I would recommend that it be used in most cases	102	34.7	29.5–40.3%
	I would recommend that it be used in an acceptable number of cases	118	40.1	34.7–45.8%
	I would recommend that it be used only in very select cases	39	13.3	9.9–17.6%
	I would recommend that it never be used	1	0.3	0.1–1.9%

*95% CI* 95% confidence interval

**Fig. 2:**
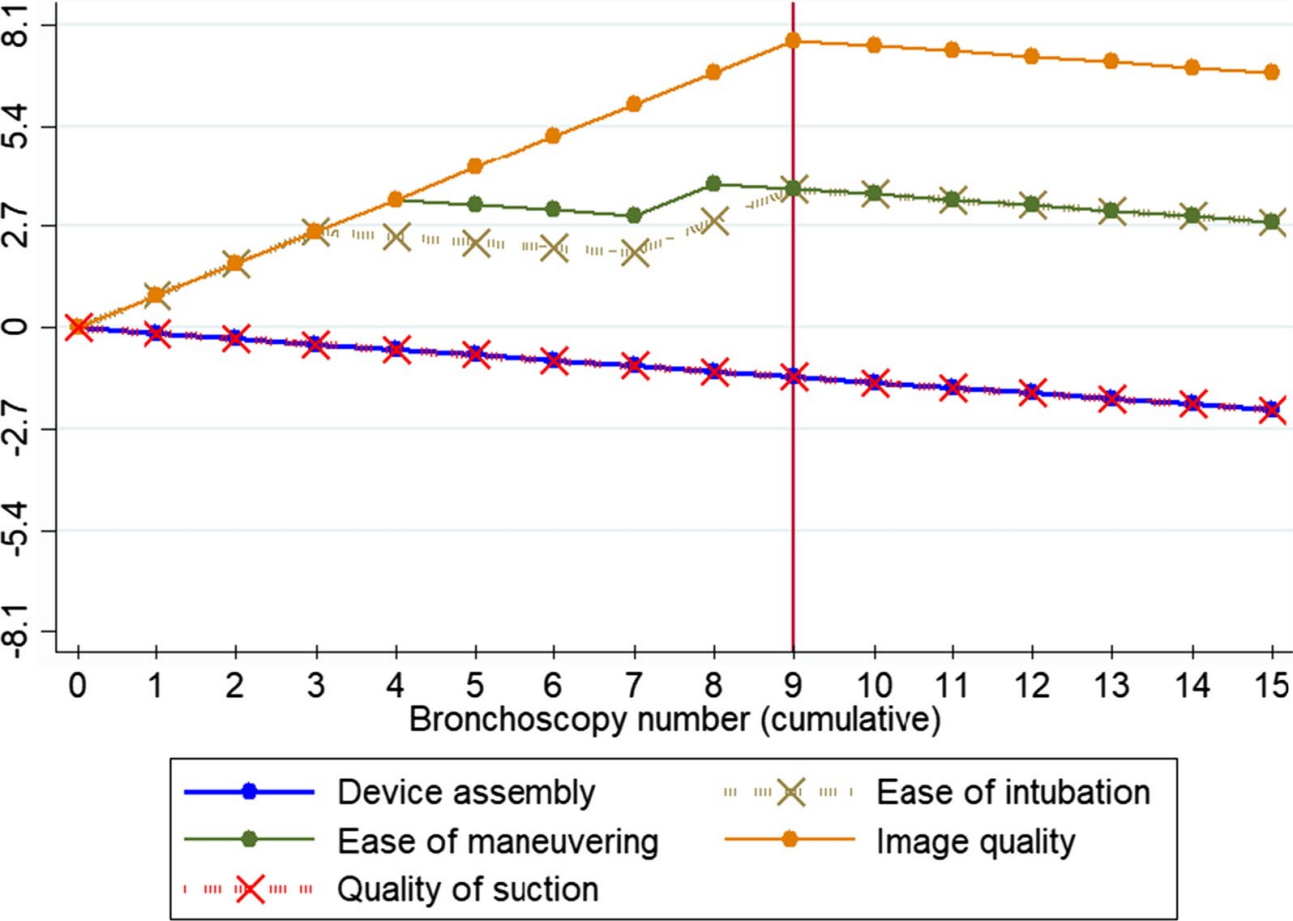
Plots of cumulative checksums (CUSUM analysis). Intubation: passing the bronchoscope through the vocal folds into the trachea. When the CUSUM curve is directed upward it indicates inadequate performance (less than 80% of procedures were scored with a standardized score ≥ 80/100), when the curve stabilizes indicates that between 80 and 90% of the procedures were rated with a standardized score ≥ 80/100, when the curve is directed downwards indicates that more than 90% of the procedures were scored with a standardized score ≥ 80/100. The assembly of the equipment and the quality of aspiration obtained standardized scores ≥ 80/100 from the first procedure. Intubation ease obtained standardized scores ≥ 80/100 in more than 80% of cases since the 3rd procedure, the ease of maneuver obtained standardized scores ≥ 80/100 in more than 80% of cases since the 4th procedure, the image quality obtained standardized scores ≥ 80/100 in more than 80% of cases since the 9th procedure

The analysis by the CUSUM Analysis graphical method to detect if there was a learning curve in the use of the Ambu^®^ aScope4™ showed the following learning points (point in which the scores exceeded 80/100 in more 80% of bronchoscopies): ease of passing the fiberoptic bronchoscope to the trachea (intubation) in the 3rd procedure, ease of maneuvering during the bronchoscopy in the 4th procedure, and image quality during the bronchoscopy in the 9th procedure (Fig. 2). Before these learning points the average scores for these aspects were between 70/100 and 80/100. The assembly of the equipment and the quality of aspiration of the bronchoscope obtained standardized scores higher than 80/100 from the first procedure.

The most outstanding characteristics of the bronchoscope were its portability and immediacy to start the procedure in 99.3% (296/300; 95% CI 97.6–99.8%), its sterility in 96.3% (287/300; 95% CI 93.5–97.9%), the possibility of taking and storing images and videos of the procedure in 99.3% of cases (298/300; 95% CI 97.6–99.8%) and 88.6% (263/297; 95% CI 84.4–91.7%) considered that the images and videos were of sufficient quality. 93% of bronchoscopists considered it useful that the bronchoscope be disposable and for single use (277/300; 95% CI 89.5–95.3%). In one of the units of non-invasive mechanical ventilation where this study was conducted, highlighted the usefulness of the aScope 4 left at the bedside of a patient who presented dyspnea due to severe accumulation of secretions, by allowing them to aspirate them under direct vision more effectively than with the aspiration probe at blind.

## Discussion

Our study provides as novel aspects the evaluation of the bronchoscopist's perception of the quality of the aScope4™ disposable bronchoscope through a standardized questionnaire and the measurement of its learning curve. The aScope4™ was very well evaluated in terms of ease of use, imaging and aspiration, obtaining an average score of 80/100 and a high degree of satisfaction in the bronchoscopist. After the 9th procedure, the scores exceeded 80/100 in more than 80% of the bronchoscopies. They highlighted its portability, immediacy to start the procedure and the possibility of storing the images.

New bronchoscopes have recently been introduced that offer advantages over existing ones. The quality assessment of these bronchoscopes should be done in the most objective way possible, to validate their functionality. The measurement of the bronchoscopist's perception using standardized questionnaires that include the most relevant domains is a key element for the validation of these devices. In the absence of a questionnaire with these characteristics, we designed one by a panel of expert bronchoscopists, which included questions related to the route of entry, ease of assembly of the equipment, ease of operation, image quality and aspiration, robustness of the equipment to maintain full functionality and to allow the planned sampling, in addition to the degree of general satisfaction.

The evaluation of the psychometric properties of the BQQ showed a very good internal consistency as measured by Cronbach's alpha, with a value of 0.88 [18]. It is noteworthy that the Cronbach's alpha coefficient can have values between 0 and 1, 0 indicates absence of consistency and 1 total consistency. Values between 0.8 and 0.9 are considered very good, values less than 0.7 are considered low and values greater than 0.94 are considered indicative of redundancy in the questions. The participation of a panel of experts in the construction of the questionnaire and the values obtained in Cronbach's alpha gave us the necessary support in aspects related to the validity of appearance, content and construct to apply the questionnaire in our study.

A single-use disposable bronchoscope has significant advantages related to reducing the risk of cross infection, ease of compliance with cleaning and disinfection regulations during non-working hours, and reducing costs related to trauma repairs during use or reprocessing of the equipment. Studies on the effectiveness of reprocessing techniques have shown failures that can occur even when current regulations are followed [4, 5]. This makes single-use bronchoscopes preferable for patients at increased risk of infection, such as immunocompromised patients, or those at risk of spreading infections by resistant or virulent germs (e.g., hepatitis B and C, HIV, multi-resistant bacteria and tuberculosis, among other). Particularly, during the current COVID-19 pandemic most respiratory societies have recommended disposable bronchoscopes to decrease transmission of the SARS-CoV-2 to other patients and to the health care providers [8–11]. However, these advantages would be of little value if the bronchoscope did not fulfill its functions with quality.

Given their high sensitivity to detect changes in positive or negative trends, the cumulative checksum graphs (CUSUM) are probably the most appropriate method to evaluate the introduction of new technologies, study learning curves and assess the quality of the results [15–17, 19]. This analysis showed that the aScope4 did not require a learning curve in aspects related to equipment assembly and aspiration quality, probably because it works similarly to reusable bronchoscopes. The disposable sheaths, also designed to reduce the risk of cross infection, had some difficulties in these aspects [20], the advantage of the single-use bronchoscope may be due to not needing to couple an external sheath with a second working channel. Image quality, ease of tracheal intubation and maneuvering had standardized scores ≥ 80/100 from the 9th procedure, with previous scores between 70/100 and 80/100, these results show a good performance of the aScope4 from the first procedure and excellent performance from the 9th procedure. However, like previous studies on the quality of single-use bronchoscopes [14, 21, 22] or disposable sheaths [20]. They did not evaluate the existence of learning curves by methods validated for this purpose, nor did they use standardized questionnaires. Their comparison with our results has these limitations. In this study 54.4% of physicians found the quality of images worse than those from reusable videobronchoscopes. Thus, reusable videobronchoscopes remain the cornerstone in interventional pulmonology units.

In a study done with a previous version of aScope4, aScope2 [21], authors observed lower image quality and greater difficulty in maneuverability. In our study, the scores in the domains related to image quality, maneuverability, aspiration, ease of assembly and general satisfaction were good, which is probably due to the technical improvements made in this new version of the device. Our study has as limitations not having included more complex procedures such as taking biopsies or punctures and not having included a control group, so that it does not allow us to establish the usefulness of aScope 4 for such procedures or the superiority or inferiority of aScope 4 versus other video bronchoscopes.

Among the advantages of the aScope 4 they highlighted the fact that it is sterile, that it is for single use, the portability and immediacy to start the procedure, the possibility of taking and storing videos and photos of the procedure. Taking and storing images can be particularly useful when the equipment is used in intensive care units, where fiberoptic bronchoscopes having such functionality are often not used, and therefore the exploration is only visualized by the bronchoscopist who performs it. The aScope 4 indications are very varied, such as emergency situations, COVID infections, mycobacteria or multi-resistant germs, immunosuppressed patients, ICU admissions, etc. This device also constitutes an advantage in the training of specialists because it allows them to visualize the examination or teach the bronchoscopic findings to the members of the medical team and could also reduce the costs related to the damage of such equipment due to the trauma they receive when entering through orotracheal tubes or non-invasive ventilation masks. In addition, the characteristics of the aScope 4 allow it to be kept permanently at the patient's bedside when there are serious problems with airway obstruction due to abundant secretions, so that they can be aspirated under direct vision in a way that is probably more effective than blindly. Finally, one of the factors to take into account in the future is the possible ecological impact of this device since it is necessary to dispose of it after use and because it is made of plastic materials.

## Conclusions

The aScope 4 scored very well in terms of ease of use, image quality, and aspiration. We observed a learning curve with excellent scores from the 9th procedure. They highlighted its portability, immediacy of use and the possibility of taking and storing images.

## Supplementary information

**Additional file 1:** CUSUM analysis.

## Data Availability

To share our data in the follow link: https://docs.google.com/spreadsheets/d/1s3TZklwXO0UX_93PbxN7Oij3OmWGhX7j1ZmhJ6IcxME/edit?usp=sharing.

## Abbreviations

CUSUM: Cumulative checksum graphs
CEA: European certification
BAL: Bronchoalveolar lavage
BAS: Bronchial aspirate
BQQ: Bronchoscope quality questionnaire
IQR: Interquartile range
