# Supplementary material for: Bronchoscopist's perception of the quality of the single-use bronchoscope (Ambu aScope4™) in selected bronchoscopies: a multicenter study in 21 Spanish pulmonology services
Source: Respir Res. 2020 Dec 2;21:320. doi: 10.1186/s12931-020-01576-w (PMC7709094; doi:10.1186/s12931-020-01576-w)
Supplement: Supplementary file 1 — Additional file 1: CUSUM analysis. [file 12931_2020_1576_MOESM1_ESM.docx]

**Cumulative checksum analysis (CUSUM Analysis)**

To assess the quality of bronchoscopies related to the number of procedures performed with the aScope4 and the existence of learning curves, we used the binary CUSUM analysis method. This type of analysis requires establishing an acceptable failure (error) rate (error level if the procedure is carried out correctly, due to the inherent variability of the test) and an unacceptable failure rate (maximum acceptable error level). We consider an acceptable failure rate of 10% (90% of the scores in the evaluated aspect ≥ 80/100) and an unacceptable failure rate of 20% (less than 80% of the scores in the evaluated aspect ≥ 80/100) ; we defined a type I error (probability of falsely accusing the bronchoscope as inadequate, designated as α) of 0,^1-3^

We build the binomial CUSUM charts in Microsoft Excel 2016 (Microsoft Corporation, Redmond, WA, USA). This method consists of the cumulative sum of failures less successes in each case (each bronchoscopy). The score for the CUSUM was calculated using the equation:

$$S_{n}=\sum(X_{i}-s)$$

Where, $S_{n}$= CUSUM, = 1 for failure and 0 for success; it is a score calculated based on the probability of acceptable and unacceptable failure rates. A score of (1 -) was added for each failure and a score of () was added for each success (that is, it was subtracted). The score (was calculated using the equation:$X_{i}ss-sss)$

$s= \frac{lnln ((1-p_{0})/ (1-p_{1}) )}{lnln ((1-p_{0})/ (1-p_{1}) )+lnln (\frac{p_{1}}{p_{0})}}$

Where $p_{0}$it was the acceptable failure rate (10%) and it was the unacceptable failure rate (20%). Therefore, using this equation = 0.1452, which means that for each failure we add 0.8548 (1- 0.1452 = 0.8548) and for each success we subtract 0.1452. We plotted the CUSUM graph by plotting the index number of each case (bronchoscopy) on the x-axis versus the cumulative sum score after that case on the y-axis. Consecutive failures drive the CUSUM curve upward while consecutive successes drive the CUSUM curve downward.$p_{1}s$

The CUSUM chart includes horizontal lines called decision limits (h1 and h0), which are the limits of an acceptable or unacceptable error rate. When the CUSUM curve crosses a decision limit (decision threshold) from above, it is inferred that the failure rates were within the predetermined acceptable rate of 10% (absence of a statistically significant difference with the predetermined acceptable failure rate of 10 %, that is, excellent performance); when the CUSUM curve crosses a decision limit from below, it is inferred that the failure rates have reached the unacceptable failure rate of 20% (absence of a statistically significant difference with the unacceptable failure rate of 20%, that is, performance inadequate); if the CUSUM curve is stable between two decision limits, continuous observation (stable performance within good levels) is indicated. Therefore, good performance is assumed when the CUSUM curve slopes downward or remains stable, but when the curve slopes upward it indicates a lower than acceptable success rate.

Decision limits (h1 and h0) were calculated based on the risk of type I errors (α) and II (β) by means of the following equations:

$$h_{1}=\frac{ln(\frac{\left( 1-\beta\right)}{\alpha})}{lnln (\frac{(1-p_{0})}{(1-p_{1}))+ln(\frac{p_{1}}{p_{0})}}}$$

$$h_{0}=\frac{-ln(\frac{\left( 1-\alpha\right)}{\beta})}{lnln (\frac{(1-p_{0})}{(1-p_{1}))+ln(\frac{p_{1}}{p_{0})}}}$$

Where $\alpha=\beta$,therefore h0 = h1 and the decision limits are multiples of h0. In our case, as$\alpha=\beta$ = 0,1; $p_{0}$= 10% and = 20%; therefore h0 = h1 = 2.71. For this reason, we mark the decision limits of our CUSUM charts as horizontal lines starting from the axis and at intervals of 2.71.$p_{1}$

**Figure S1. Indications for use of the bronchoscope**



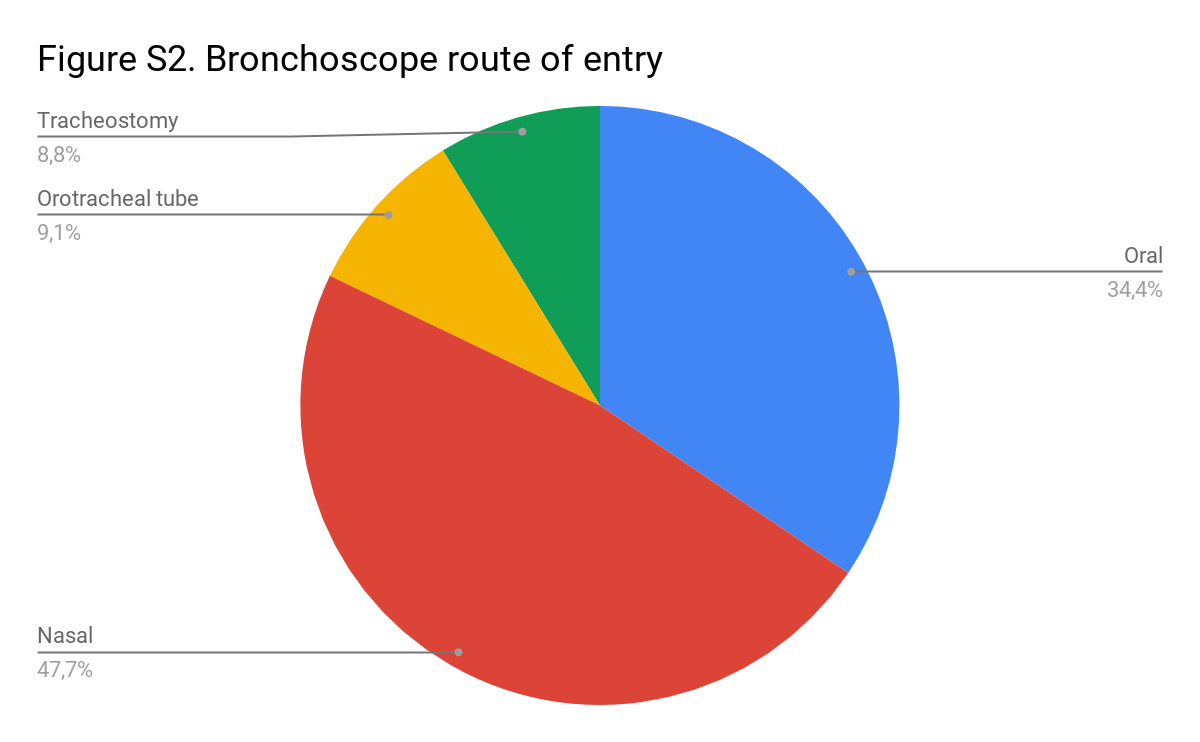


**Table S1 Average quality of the Ambú aScope4TM bronchoscope according to the number of fiberoptic bronchoscopes**

| FBC No. | Ease of mounting the equipment | Ease of mounting standardized equipment | Intubation ease | Ease in standardized intubation | Ease of maneuvering | Ease of maneuver standardized | Global image quality | Standardized global image quality | Global aspiration quality | Standardized global aspiration quality |
| --- | --- | --- | --- | --- | --- | --- | --- | --- | --- | --- |
| 1.0 | 4.4 | 87.5 | 7.8 | 78.3 | 7.7 | 77.3 | 7.2 | 72.4 | 8.0 | 80.0 |
| 2.0 | 4.5 | 90.0 | 7.9 | 79.4 | 7.9 | 78.9 | 7.5 | 75.0 | 8.1 | 81.1 |
| 3.0 | 4.5 | 90.0 | 7.9 | 79.3 | 7.9 | 79.4 | 7.5 | 74.7 | 8.2 | 81.9 |
| 4.0 | 4.5 | 90.0 | 8.3 | 82.7 | 7.8 | 78.5 | 7.4 | 74.2 | 8.1 | 81.2 |
| 5.0 | 4.5 | 89.6 | 8.4 | 83.9 | 8.2 | 82.0 | 7.5 | 75.2 | 8.1 | 81.2 |
| 6.0 | 4.6 | 91.2 | 8.4 | 84.1 | 8.1 | 81.2 | 7.4 | 74.0 | 8.2 | 82.0 |
| 7.0 | 4.5 | 90.9 | 8.2 | 82.4 | 8.1 | 80.9 | 7.4 | 73.5 | 8.1 | 81.0 |
| 8.0 | 4.6 | 91.8 | 7.8 | 77.6 | 7.5 | 75.3 | 7.1 | 71.3 | 8.5 | 84.7 |
| 9.0 | 4.5 | 90.0 | 7.9 | 79.3 | 8.1 | 80.7 | 7.5 | 74.6 | 8.3 | 82.9 |
| 10.0 | 4.5 | 90.0 | 8.8 | 87.5 | 8.4 | 84.2 | 8.0 | 80.0 | 8.7 | 86.7 |
| 11.0 | 4.3 | 86.0 | 9.0 | 90.0 | 8.7 | 87.0 | 8.3 | 83.0 | 8.8 | 88.0 |
| 12.0 | 4.7 | 93.3 | 8.8 | 87.8 | 8.7 | 86.7 | 8.1 | 81.1 | 8.8 | 87.8 |
| 13.0 | 4.4 | 88.0 | 9.0 | 90.0 | 8.8 | 88.0 | 8.5 | 85.0 | 9.0 | 90.0 |
| 14.0 | 4.2 | 84.0 | 8.8 | 88.0 | 8.6 | 86.0 | 8.2 | 82.0 | 8.6 | 86.0 |
| 15.0 | 4.0 | 80.0 | 8.0 | 80.0 | 8.0 | 80.0 | 8.0 | 80.0 | 8.0 | 80.0 |

Notes: BCF: fiberoptic bronchoscopy

**REFERENCES**

1. Bolsin S, Colson M. The use of the Cusum technique in the assessment of trainee competence in new procedures. International journal for quality in health care: journal of the International Society for Quality in Health Care. 2000; 12: 433-8. doi:

2. Kemp SV, El Batrawy SH, Harrison RN, Skwarski K, Munavvar M, Rosell A, et al. Learning curves for endobronchial ultrasound using cusum analysis. Thorax. 2010; 65: 534-8. doi: 10.1136 / thx.2009.127274

3. Eltoum IA, Chhieng DC, Jhala D, Jhala NC, Crowe DR, Varadarajulu S, et al. Cumulative sum procedure in evaluation of EUS-guided FNA cytology: the learning curve and diagnostic performance beyond sensitivity and specificity. Cytopathology: official journal of the British Society for Clinical Cytology. 2007; 18: 143-50. doi: 10.1111 / j.1365-2303.2007.00433.x
